# Vertical Flow Immunoassay Based on Carbon Black Nanoparticles for the Detection of IgG against SARS-CoV-2 Spike Protein in Human Serum: Proof-of-Concept

**DOI:** 10.3390/bios13090857

**Published:** 2023-08-29

**Authors:** Maria Kropaneva, Pavel Khramtsov, Maria Bochkova, Sergey Lazarev, Dmitriy Kiselkov, Mikhail Rayev

**Affiliations:** 1 Institute of Ecology and Genetics of Microorganisms, Ural Branch of Russian Academy of Sciences, 614081 Perm, Russia; kropanevamasha@gmail.com (M.K.); mraev@iegm.ru (M.R.); 2 Biology Faculty, Perm State University, 614990 Perm, Russia; 3 Institute of Technical Chemistry, Ural Branch of Russian Academy of Sciences, 614013 Perm, Russia

**Keywords:** SARS-CoV-2, COVID-19, vaccination, antibody, vertical flow assay, paper-based assay, carbon black nanoparticles

## Abstract

Point-of-care tests play an important role in serological diagnostics of infectious diseases and post-vaccination immunity monitoring, including in COVID-19. Currently, lateral flow tests dominate in this area and show good analytical performance. However, studies to improve the effectiveness of such tests remain important. In comparison with lateral flow tests, vertical flow immunoassays allow for a reduction in assay duration and the influence of the hook effect. Additionally, the use of carbon black nanoparticles (CNPs) as a color label can provide a lower detection limit (LOD) compared to conventional colloidal gold. Therefore, we have developed a vertical flow immunoassay for the detection of IgG against SARS-CoV-2 spike protein in human serum samples by applying a conjugate of CNPs with anti-human IgG mouse monoclonal antibodies (CNP@MAb). The vertical flow assay device consists of a plastic cassette with a hole on its top containing a nitrocellulose membrane coated with spike protein and an absorbent pad. The serum sample, washing buffer, and CNP@MAb flow vertically through the nitrocellulose membrane and absorbent pads, reducing assay time and simplifying the procedure. In positive samples, the interaction of CNP@MAb with anti-spike antibodies leads to the appearance of black spots, which can be visually detected. The developed method allows for rapid visual detection (5–7 min) of IgG vs. spike protein, with a LOD of 7.81 BAU/mL. It has been shown that an untrained operator can perform the assay and visually evaluate its results. Thus, the presented assay can be used in the further development of test systems for the serological diagnostics of COVID-19 or post-vaccination immunity monitoring.

## 1. Introduction

The pandemic associated with the novel beta-coronavirus (β-CoVs or Beta-CoVs) severe acute respiratory syndrome coronavirus 2 (SARS-CoV-2), which caused the 2019 outbreak of coronavirus disease (COVID-19), has become a major public health problem [1]. This has led to the rise of many studies devoted to the development of test systems for the serological diagnostics of COVID-19 and post-vaccination immunity monitoring [2,3]. However, the development of point-of-care tests for limited resource settings still remains important [4]. Such assays should be easy to use, stable during storage, and available for large-scale production. Currently, lateral flow tests dominate in this area, showing high sensitivity and specificity, which can reach 97–99% [5]. Despite this, lateral flow assays from a number of manufacturers did not show sufficient effectiveness, and their production and sales were suspended by regulators [6,7,8].

**Figure 1:**
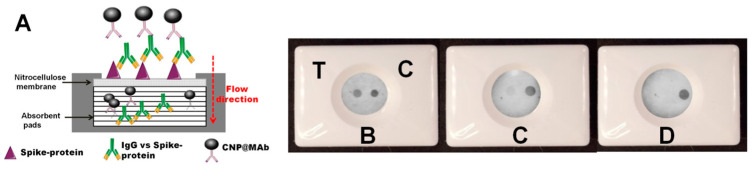
(**A**) Scheme of the VFIA for IgG vs. spike protein detection. Examples of VFIA results: (**B**) VFIA results of a serum sample with high IgG vs. spike protein levels; (**C**) VFIA results of a serum sample with a medium IgG vs. spike protein level; (**D**) VFIA results of a serum sample with a low IgG vs. spike protein level or a negative serum sample. C–control; T–test.

The vertical flow immunoassay (VFIA), also known as immunofiltration or the flow-through immunoassay, is a point-of-care test that consists of a matchbox-sized plastic cassette containing an absorbent pad and a porous nitrocellulose membrane on its top. The plastic lid of the cassette has an injection hole which is used for the addition of samples and reagents. Capture and recognition of the analyte occur on the surface of the nitrocellulose membrane, while excess reagents pass through the membrane into the absorbent pad (Figure 1A). The application of colored labels, usually colloidal gold, allows for visual detection of the analyte (Figure 1B–D), although scanners or cameras can be used to obtain quantitative results.

The vertical flow immunoassay is used for the detection of biomarkers [9,10], antibodies [11,12,13], pathogenic organisms [14,15], and antibiotics [16]. It is worth mentioning that the Canadian company bioLytical^®^ Laboratories produces a test for the determination of antibodies against HIV in a flow-through format [17]. The test was approved by the WHO and the FDA. In comparison with lateral flow tests, the VFIA allows for a reduction in assay time and the elimination of a hook effect, as demonstrated by Oh and co-authors [18,19,20]. The most frequently utilized labels in VFIAs are colloidal gold [11,12,15,20,21,22,23] and horseradish peroxidase [14,15,16,17,18,19,20,21,22,23,24]. Quantum dots [9] and colloidal dyes [25] can also be used as labels.

Colloidal carbon, as a colored label, provides numerous advantages in the development of colorimetric assays. Carbon black conjugates are intensely colored, allowing for a high level of analytical signals and a high signal-to-noise ratio, which can significantly decrease the limit of detection. For example, Porras and co-authors have demonstrated that carbon nanoparticles (CNPs) provide higher sensitivity (3.8 times) compared to gold nanoparticles in lateral flow tests for nucleic acid detection [26]. The details of CNP usage in immunoassays are discussed in depth in previous studies [27,28,29,30]. Moreover, carbon black is a widely used and inexpensive material, with a standardized production process and properties. A number of research groups have taken advantage of CNPs for the development of paper-based and lateral flow tests [31,32,33,34]. Thus, the application of carbon nanoparticle conjugates in VFIAs is a promising option.

Herein, we have developed, for the first time, a vertical flow immunoassay based on carbon black nanoparticles for the detection of immunoglobulin G against the spike protein of SARS-CoV-2. The immunoassay involves several simple steps and can be performed in modern laboratories by untrained operators (CV for intra-operator precision does not exceed 15%). The detection limit of the assay is 7.81 BAU/mL. Visual detection of IgG vs. the spike protein in a single serum sample can be assessed within 5–7 min. The work also presents and discusses the methodological aspects of the developed assay.

## 2. Materials and Methods

### 2.1. Materials

Streptavidin was obtained from ProspecBio (Ness-Ziona, Israel). Tween-20 was purchased from ITW (Milano MI, Italy). Mouse monoclonal IgG2a against human IgG, further designated as Mab, and the recombinant spike protein of SARS-CoV-2 were obtained from HyTest (Turku, Finland). Human IgG from human serum and casein was obtained from Sigma-Aldrich (Saint Louis, MO, USA). Bovine serum albumin was acquired from Biosera (Cholet, France). Biotinylation of BSA was performed as described in [35].

Samples of different carbon black types (N115, N231, N326, N330, N772, P803) were kindly provided by A. L. Gabov (Perm State University, Russia; Chemistry faculty, Department of Physical Chemistry) as a dry powder.

Instrumentation: the Multiskan Sky UV-Vis Reader was obtained from Thermo Scientific (Waltham, MA, USA). The ZetaSizer NanoZS particle analyzer was acquired from Malvern (Malvern, UK). A VCX-130 ultrasonic processor was obtained from Sonics & Materials (Newtown, CT, USA). The scanning electron microscope used was an FEI Quanta 650FEG (Thermo Scientific, Waltham, MA, USA).

Components of devices for vertical flow immunoassays: nitrocellulose membranes (CLW-040, 0.3 µm/0.45 μm/0.8 μm), AP-080 absorbent pads, and plastic cases were from acquired from Advanced Microdevices Pvt. Ltd. (MDI) (Ambala Cantt, India).

Buffers for preparation of carbon black nanoparticle conjugates:

Borate buffer (pH 8.8 (BB)) was prepared by adjusting 50 or 100 mM H_3_BO_3_ solutions (Sigma-Aldrich, Saint Louis, MO, USA) to the desired pH with 0.1 M NaOH (Panreac, Barcelona, Spain). Coupling buffer: 50 mM BB; washing buffer: 50 mM BB + 1% (*w*/*v*) BSA; storage buffer: 100 mM BB + 1% (*w*/*v*) BSA + 0.53% ProClin950 (0.05% 2-methyl-4-isothiazolin-3-on) (Sigma-Aldrich, Saint Louis, MO, USA).

Buffers for vertical flow immunoassays:

Phosphate-buffered saline (PBS), 0.01 M: 0.137 mol/L NaCl + 0.0027 mol/L KCl, pH 7.4 (Ecoservice, Moscow, Russia) + 0.53% ProClin950. Coating buffer: PBS; washing buffer: PBS + 0.1% Tween-20; blocking buffer: PBS + 0.4% Tween-20.

All buffers were prepared using deionized water.

### 2.2. Methods

#### 2.2.1. Conjugation of CNPs with Anti-Human MAb and Bi-BSA

Carbon black nanoparticles (CNPs) were conjugated with MAb or Bi-BSA according to the method described in [27], with modifications. CNPs were diluted to the final concentration of 2 mg/mL with 1 mL of coupling buffer and ultrasonicated in an ice bath (probe diameter—3 mm; amplification—60%; duration—1 min). Then, the required amount of Bi-BSA or MAb was added, and the mixture ultrasonicated again (probe diameter—3 mm; amplification—60%; duration—1 min). After incubation for 60 min at 37 °C on a rotary mixer (10 rpm), BSA was added to the final concentration of 2% and incubated (1 h, 37 °C, rotary mixer). After this stage, the absorbance at 450 nm of obtained suspensions was measured and used to assess nanoparticle concentration at the following synthesis stages. Next, the resulting suspensions of CNP@Mab or CNP@Bi-BSA were precipitated by centrifugation at 20,000× *g* for 30 min, and the resulting pellets were resuspended in washing buffer before being briefly sonicated (10 s, 60%) and centrifuged at 20,000× *g* for 15 min. After the third wash cycle, the resulting pellets were resuspended in a 0.5 mL storage buffer and ultrasonicated using a probe sonicator in an ice bath (probe diameter—3 mm; amplification—60%; duration—1 min). The conjugates were stored at 4 °C and briefly sonicated before usage.

#### 2.2.2. CNP Conjugates Characterization

The size and monodispersity of nanoparticles were measured by dynamic light scattering (DLS). For this, nanoparticles were diluted 1:350 in deionized water.

To assess the absorbance at 450 nm of the carbon black nanoparticles, obtained suspensions were diluted 1:101 in the washing buffer (10 μL of particles + 1000 washing buffer) in a glass cuvette.

To obtain the SEM images of nanoparticles, CNP conjugate samples were dropped onto silicon wafers (5 × 5 mm), dried overnight at room temperature, and analyzed by SEM.

#### 2.2.3. Preparation of Vertical Flow Immunoassay Devices

The nitrocellulose membrane was coated with spike protein (2 µL per dot) diluted in coating buffer and dried (30 min RT, 90 min at 37 °C in a drying oven). Next, the immunosorbent was transferred to the plate and washed using 30 mL of washing buffer for 5 min 3 times. After that, the membrane was incubated with 35 mL of blocking buffer (60 min, 37 °C) for elimination of the nonspecific interactions. The washing procedure was then repeated. After that, the immunosorbent was dried (30 min RT, 90 min at 37 °C in a drying oven), cut into small strips (15 mm and 15 mm), and placed over 10 stacks of absorbent pads supported on a solid plastic case. Following this, the presented device was closed using a lid with a hole for sample addition (Figure S1).

#### 2.2.4. Assay Procedure

**Figure 2:**
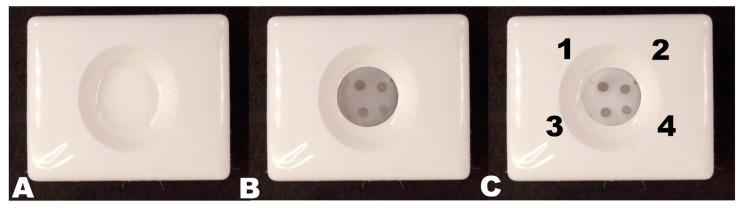
Vertical flow assay procedure. (**A**) Addition of the analyzed sample; (**B**) addition of CNP@ MAb; (**C**) addition of 200 μL washing buffer. Spike protein concentration: (1) 0.5 mg/mL; (2) 0.25 mg/mL; (3) 0.125 mg/mL; (4) 0.0625 mg/mL.

The reagents were dropped onto the immunosorbent sequentially. Initially, 150 μL of washing buffer was dropped onto the immunosorbent to the full absorption of liquid, followed by the addition of 100 μL of test sera in blocking buffer (Figure 2A). After the 1 min incubation and second wash cycle, 80 μL of CNP@MAb was added to visualize the spot on the immunosorbent and incubated for 1 min (Figure 2B). Assay results were analyzed after the third wash cycle with 200 μL of washing buffer (Figure 2C). For optimization experiments, the images of the immunosorbent were processed using ImageJ software according to the method described in [36]. A detailed description is provided in the Supplementary Materials Section (Figure S4).

#### 2.2.5. Assay Parameters for Direct Detection of Streptavidin

**Figure 3:**
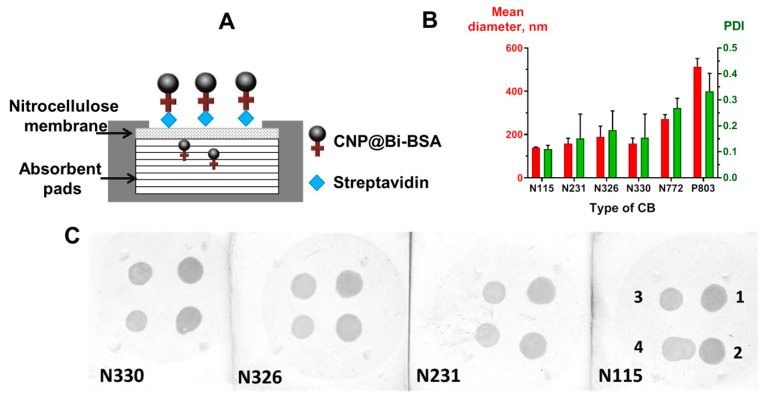
(**A**) Scheme of the VFIA for direct streptavidin detection. Results of carbon black type optimization: (**B**) mean diameter and polydispersity of the obtained CNP@Bi-BSA (PDI—polydispersity index; the vertical bars indicate the standard deviation, n = 3); (**C**) functional activity of the obtained CNP@Bi-BSA (streptavidin concentrations in mg/mL: 1–1, 2–0.5, 3–0.25, 4–0.125).

The nitrocellulose membranes coated with streptavidin at four different concentrations (0.5, 0.25, 0.12, 0.06 mg/mL) were first dried and washed. Next, the membranes were dipped into a reservoir with a blocking buffer and incubated for 1 h at 37 °C. After washing and drying, prepared nitrocellulose membranes were used in VFIAs for streptavidin detection. Analytical signals were obtained using CNP@BSA-Bi diluted to a final concentration of 0.25 mg/mL in the blocking buffer (Figure 3A).

#### 2.2.6. Assay Parameters for Indirect Detection of IgG vs. Spike Protein

Spike protein was adsorbed onto the nitrocellulose membrane at a final concentration of 0.25 mg/mL, while analyzed serum samples were dissolved 1/10 in the blocking buffer. In the blocking solution, 0.25 mg/mL CNP@MAb was used to obtain analytical signals (Figure 1A).

#### 2.2.7. Clinical Serum Samples

Human serum samples from patients with a verified diagnosis of new coronavirus infection (COVID-19) were obtained from Clinical Industrial Hospital №1, Perm Krai, Russia. Due to the widespread nature of new coronavirus infections and vaccination, obtaining negative serum samples is problematic. Therefore, serum samples obtained before 2019 were used as negative samples. All clinical samples were first analyzed and the levels of immunoglobulin against SARS-CoV-2 spike protein (IgG vs. spike protein) were measured using a commercial ELISA kit (Vector-Best, Russia, www.vector-best.ru, accessed on 4 May 2023). The test was performed according to the manufacturer’s instructions. As a result, positive blood sera with various concentrations of IgG vs. spike protein were collected. Samples obtained before 2019 according to the results of the ELISA were determined as negative.

Additionally, positive and negative serum pools were prepared by mixing 3 positive serum samples and 10 negative serum samples, respectively. The preserving agent Proclin 950 was added in each pool to a final concentration of 0.53%. The concentration of IgG vs. spike protein was 5536.2 BAU/mL in the positive serum pool and 0 BAU/mL in the negative serum pool, according to ELISA.

This research was performed according to the World Medical Association’s Declaration of Helsinki and the Council of Europe protocol on the Convention of Human Rights and Biomedicine and was approved by the Ethics Committee of the Institute of Ecology and the Genetics of Microorganisms, Ural Branch of the Russian Academy of Sciences (IRB00010009). Written informed consent was obtained from all the participants.

## 3. Results and Discussion

### 3.1. Optimization of the Preparation of CNP Conjugates

#### 3.1.1. Carbon Black Type Optimization

Different types of carbon black have a wide range of primary particle sizes, surface areas per unit of mass, and degrees of particle aggregation. In terms of immunoassay development, particle size and surface characteristics contribute to tinting strength, blackness, and the protein adsorption process [37]. These parameters can ultimately affect the specificity and sensitivity of the immunoassay, as well as the stability of the conjugate [38].

In this series of experiments, for reasons of economy, CNPs were functionalized with Bi-BSA (CNP@Bi-BSA) rather than with monoclonal antibodies. We obtained CNP@Bi-BSA based on six types of carbon black: N115, N231, N326, N330, N772, and P803. The size and polydispersity of CNP@Bi-BSA were measured, as well as performance in a model VFIA for direct streptavidin detection (Figure 3A). As expected, the type of carbon black affected the size of the conjugates. The application of carbon black N115 provided CNP@Bi-BSA with the smallest diameter of 139.9 ± 1.63 nm and a polydispersity index of 0.11 ± 0.01 (Figure 3C). Carbon blacks N772 and P803 yielded very large conjugates that could not pass through the membrane and were excluded from further comparative analysis. For other CNP@Bi-BSA, the diameters and PdI values were in the range of 157–188 and 0.11–0.11, respectively. It was shown that the functional activity of CNP conjugates did not depend on the type (N115, N231, N326, N330) of carbon black (Figure 3B). In further studies, carbon black N115 was used because it has the lowest size and polydispersity.

#### 3.1.2. The Optimal Amount of MAb

We conjugated CNPs with MAb in ratios ranging from 10 to 250 μg of MAb per 1 mg of CNP and compared the obtained CNP@MAb in the vertical flow immunoassay of IgG vs. spike protein (Figure 1A). The conjugate comprising CNPs with bovine serum albumin in the ratio of 250:1 was used as a negative control.

**Figure 4:**
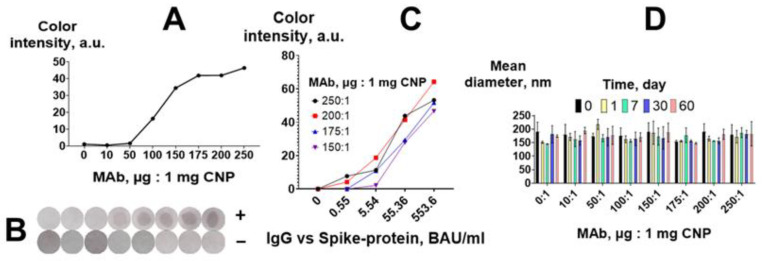
Optimization of CNP@MAb preparation. (**A**) Analytical signal of the VFIA for a positive serum pool; (**B**) visual assessment of the VFIA for a positive (+) and negative (−) serum pool; (**C**) calibration curves of IgG vs. spike protein obtained in the VFIA; (**D**) colloidal stability of CNP@MAb conjugates (mean ±SD, n = 3).

The vertical flow assay was performed using a pool of positive sera (5536.2 BAU/mL) and negative sera (0 BAU/mL). After the completion of the assay, the membranes were removed, dried, and scanned, and the obtained images were processed using ImageJ software. The analytical signal increased as the MAb-to-CNP ratio increased (Figure 4A,B), and a substantial increment in the signal was observed from the ratio of 100:1 to the ratio of 150:1. No colored spots were observed in either the negative samples or the control conjugate.

The performance of the four best CNP@MAb conjugates (with MAb-to-CNP ratios from 150:1 to 250:1) was compared using 10-fold dilutions of the positive pool. The final concentration of IgG vs. spike protein in the diluted samples was 0.55, 5.54, 55.4, and 553.6 BAU/mL. The pool of negative sera was used as a zero sample. The highest signal was obtained when the MAb-to-CNP ratios were 200:1 (Figure 4C).

The amount of protein on the CNP surface can affect its size and polydispersity. Insufficient protein coating can decrease the stability of the conjugates and, accordingly, the efficiency of the assay [39,40,41,42]. According to DLS, the mean diameter of the obtained CNP@MAb was 150–170 nm (Figure 4D). SEM images showed that the nanoparticles had a spherical shape (Figure S2). The size of the CNP@MAb was approximately the same for all MAb-to-CNP ratios and did not change for 2 months at 4 °C (Figure 4D). Thus, the MAb-to-CNP ratio of 10 μg:1 mg is sufficient to maintain the colloidal stability of the obtained nanoparticles. However, from the point of view of VFIA development, a ratio of 200 μg of MAb per 1 mg of CNP is optimal.

#### 3.1.3. Reproducibility of CNP@MAb Preparation

**Figure 5:**
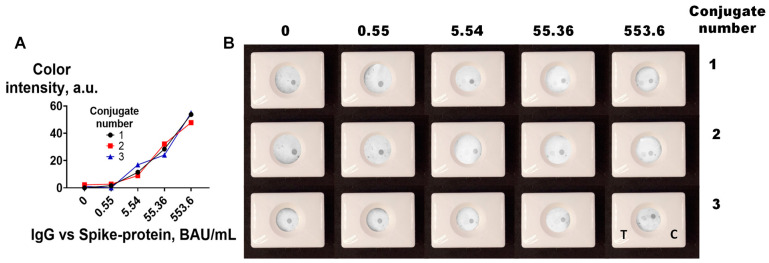
Reproducibility of CNP functionalization: (**A**) Results obtained by scanner and ImageJ processing. (**B**) Visual assessment of VFIA. T–test; C–control.

The reproducibility of nanoparticle conjugate synthesis is essential for its further practical application. To assess the reproducibility of the functionalization, three batches of CNP@MAb were prepared. A VFIA for IgG vs. spike protein determination was constructed using positive pooled serum diluted tenfold in blocking buffer, and negative pooled serum was used as a zero sample for assessment of the functional activity of CNP@MAb. Human IgG and spike protein at a concentration of 0.25 mg/mL were dotted in the control and test zone, respectively. The results are shown in Table S1 and Figure 5. Nanoparticles with similar sizes (183–184 nm), polydispersity (0.17–0.19), concentrations (3.3–4.2), and functional activities were obtained.

### 3.2. Optimization of the Vertical Flow Immunoassay

#### 3.2.1. Membrane Type Optimization

**Figure 6:**
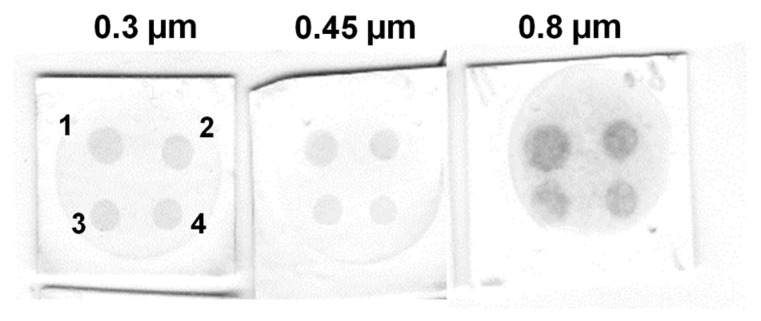
Influence of nitrocellulose membrane pore size on the results of a model VFIA based on CNP@Bi-BSA. Streptavidin concentration: (1) 1 mg/mL; (2) 0.5 mg/mL; (3) 0.25 mg/mL; (4) 0.125 mg/mL.

It is known that the pore size of the nitrocellulose membrane can affect the results of solid phase immunoassays. For example, a small pore size provides a larger surface area and a greater number of antigen-binding sites, resulting in lower limits of detection [43]. On the other hand, small pore size can lead to a decrease in sensitivity due to the high background signal. Furthermore, a small pore size can impede the flow rate, consequently impacting the assay results and its time [44]. Thus, nitrocellulose membranes with pore sizes of 0.3, 0.45, and 0.8 µm were tested in the model VFIA for streptavidin detection (Figure 3A). A pore size of 0.3 μm provides a high analytical signal, low background, and uniformly colored spots (Figure 6).

#### 3.2.2. Optimal Blocking Solution

The purpose of blocking a nitrocellulose membrane is to prevent nonspecific interactions between assay components. Blocking also serves other functions, including the maintenance of membrane hydration, the modification of wicking rates, and the stabilization of adsorbed proteins [44,45]. The concentration and type of blocking agent are usually determined empirically for compatibility with the specific sample and antibody–antigen system.

**Figure 7:**
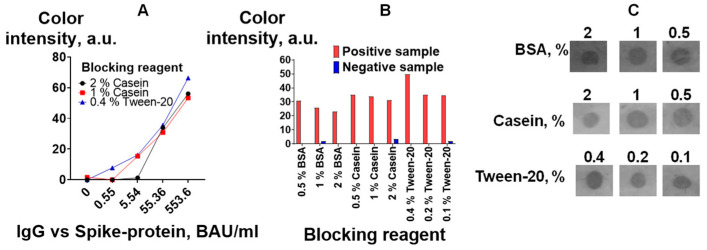
Optimization of blocking buffers: (**A**) calibration curves for the determination of IgG vs. spike protein according to the VFIA. Analytical signals of the vertical flow-through assay: results of image analysis with ImageJ software (**B**) and visual assessment of positive samples (**C**).

The effect of various common blocking agents (casein, BSA, and detergent Tween-20) [45,46] at three different concentrations was assessed by detecting IgG vs. spike protein. Figure 7B,C show that Tween-20 at a concentration of 0.4% provides the highest signal and a low background. It should be noted that the level of the background signal was lower when casein was used (Figure 7C). This could affect the assay results, especially for samples with low antibody levels. Therefore, the influence of the three best blocking solutions on the calibration curves in the IgG vs. spike protein assay was studied. Tween-20 at a concentration of 0.4% provides better detection limits (the color of the test zone for an antibody concentration of 0.55 BAU/mL could be distinguished from the color of the test zone without antibodies) (Figure 7A). In further studies, 0.4% Tween-20 was used for blocking and dilution of the analyzed samples and the detection reagent.

#### 3.2.3. Optimal Concentrations of Spike Protein and CNP@MAb

**Figure 8:**
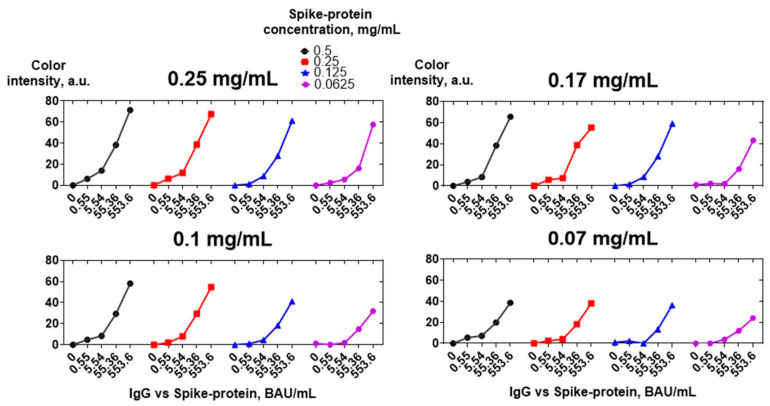
Optimization of spike protein and CNP@MAb concentrations. Concentrations of CNP@MAb are specified above the graphs.

Spike protein was adsorbed on a nitrocellulose membrane at concentrations of 0.5, 0.25, 0.125, and 0.0625 mg/mL. Tenfold dilutions of the positive sera pool were analyzed. Bound IgG vs. spike protein was detected using four concentrations of CNP@MAb: 0.25, 0.17, 0.1, and 0.07 mg/mL. Figure 8 shows that a concentration of 0.25 mg/mL is optimal for both spike protein and CNP@MAb. A 0.5 mg/mL concentration of spike protein can also be used, but no essential differences in sensitivity compared to a concentration of 0.25 mg/mL were observed. A reduction in the concentration of both the spike protein and CNP@MAb leads to a significant decrease in the analytical signal in samples with low antibody levels (0.55 and 5.54 BAU/mL).

#### 3.2.4. Optimization of an Assay Procedure

**Figure 9:**
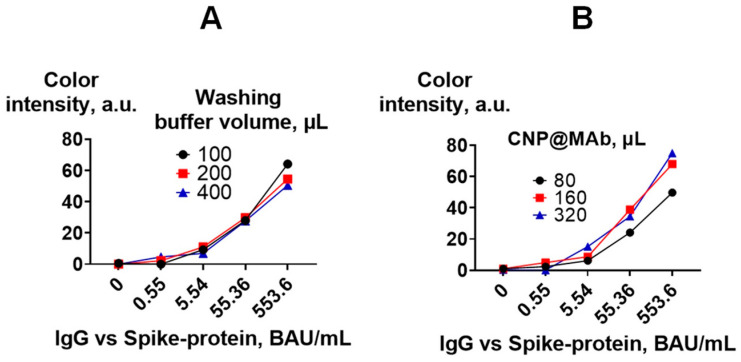
Optimization of the VFIA procedure. Calibration curves for the determination of IgG vs. spike protein are presented. (**A**) Optimal washing buffer volume; (**B**) optimal CNP@MAb volume.

The volume of the CNP@MAb and washing buffer at the last stage of the assay (washing step after incubation with CNP@MAb) was optimized. The optimal volume of CNP@MAb was found to be 80 µL. Increasing the volume of the detection reagent to 320 µL did not significantly affect the analytical signal of samples with low antibody levels (0.55 BAU/mL) (Figure 9B). The volume of the washing buffer also did not affect the assay results (Figure 9A).

### 3.3. Assay Validation

**Figure 10:**
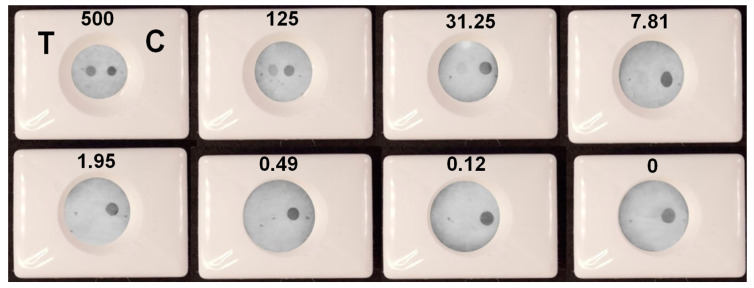
Determination of IgG vs. spike protein by the VFIA in optimal conditions. Concentrations of antibodies in BAU/mL are specified above the images. T—test, C—control.

A VFIA for IgG vs. spike protein detection was constructed under optimal experimental conditions using negative pooled serum diluted tenfold in blocking buffer as a diluent for analyzed samples. Human IgG and spike protein at a concentration of 0.25 mg/mL were dotted in the control and test zone, respectively. The assay was performed in triplicate for each antibody concentration (Figure S5A). It can be seen that when the concentration of IgG vs. spike protein is 7.81 BAU/mL or higher, the color of the test zone can be distinguished from the background (Figure 10). Thus, the limit of detection (LOD) of the presented VFIA was determined to be 7.81 BAU/mL (this parameter was determined in the same way as it was in [23], which was dedicated to the development of a VFIA for visual result assessment). This result was confirmed by the result assessment obtained using Image J software (Figure S5B). Taking into account that the analyzed sample is diluted 1/10 during the VFIA process, the developed vertical flow carbon black immunoassay allows for rapid visual identification of samples with antibody concentrations greater than 78.1 BAU/mL.

For the assessment of assay reproducibility, ten serum samples with different concentrations of IgG vs. spike protein were tested in six replicates. The serum samples were diluted tenfold with a blocking buffer before the assay. Processing the assay results using ImageJ software showed that the coefficients of variation did not exceed 25% for serum samples containing IgG vs. spike protein in concentrations ranging from 5473.09 to 108 BAU/mL and higher. For negative serum samples and two samples with antibody concentrations lower than 55 BAU/mL, the coefficient of variation exceeded 100% (Table 1).

This high coefficient of variation can be attributed to the measurement of background intensity, which is relatively non-uniform, rather than the spots.

For the validation of visual assessment, the sensitivity and specificity rates were determined. These parameters are often used to evaluate qualitative analytical methods [47]. In this study, specificity rate indicated the assay’s ability to determine all six replicates of one negative sample as negative, while sensitivity rate indicated the assay’s ability to determine all six replicates of one positive sample as positive. Visual assessment was performed for all six replicates of each serum sample. Positive results were marked as 1, and negative results were marked as 0, as shown in Table 2. The sensitivity and specificity rates were then calculated for each serum sample. The values are expressed as percentages. We expected that serum #33 and #48 with concentrations below 55 BAU/mL would be identified as negative, because antibody concentrations in these samples after dilution were lower than the detection limit. It was confirmed that the specificity rate was 100% for samples with antibodies below the LOD and two truly negative samples.

It was determined that five positive serum samples with antibody concentrations ranging from 5473.09 to 359.3 BAU/mL demonstrated a 100% sensitivity rate. All six replicates for these serums were determined as positive. One replicate for a sample with an antibody level near the detection limit (#58, 108 BAU/mL) was defined as negative, resulting in a sensitivity rate of 83%.

The evaluation showed that the developed method can determine positive serum as positive with more than 95% reliability for antibody concentrations above 359.3 BAU/mL. The performed VFIA allows for the detection of negative serum as negative with a 100% specificity rate.

To determine inter-operator precision, two blood serum samples (negative and highly positive) were analyzed by eight different operators using the optimized VFIA. The operators had various levels of experience in assay performance and were not aware of the sample status. Each operator received written step-by-step instructions for the VFIA procedure, as well as tubes with samples, diluents, conjugate, and washing buffer. The assay results were visually assessed according to the following scheme: two spots indicating the result is positive (1), one spot in the control zone indicating the result is negative (0), and one spot in the test zone or no spots indicating the test is invalid (Figure S3). Additionally, the results of the assay were processed using Image J software.

**Figure 11:**
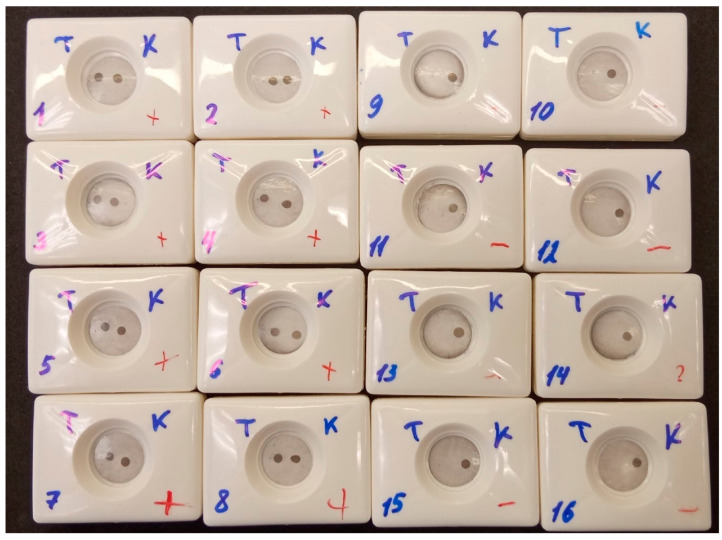
Inter-operator precision. T—test; K—control (Russian); 1–8—positive sample; 9–16—negative sample. Each operator performed tests with positive and negative samples: operator 1—cassettes 1 and 9, operator 2—cassettes 2 and 10, and so on.

All operators rated the result of analysis of the positive serum sample as positive (Figure 11). The coefficient of variation for positive results, as assessed with Image J software, was 10.44%, which is within acceptable limits (Table S2) [48]. Eight operators rated the result of analysis of the negative serum sample as negative, but one operator (Figure 11, cassette 9) rated the negative sample as positive. This false-positive result was confirmed by analysis of the membrane with Image J software (Table S1). This could be due to an error during the assay procedure, as the operator was unsure if they changed pipette tips when applying positive and negative samples. It should also be noted that another operator (Figure 11, cassette 14) had doubts about the results of the visual assessment of the negative sample. This can be explained by the non-uniform background.

## 4. Conclusions

In this study, we have demonstrated, for the first time, the development process of a nitrocellulose-based vertical flow immunoassay for the rapid visual qualitative detection of IgG versus spike protein in human serum samples using a CNP-based conjugate as a detection reagent. The immunoassay principle demonstrated herein can be applied to create VFIAs for the detection of antibodies against various infectious diseases. The optimized assay requires only a few minutes and can be performed by personnel with limited experience in immunoassays. As stated in the title of this paper, we consider this work to be a proof-of-concept study. Determining total IgG against spike protein content has little clinical significance, as there is no definite protective concentration [49]. Therefore, we consider the developed immunoassay rather as a platform for the construction of VFIAs for neutralizing antibodies, which disrupt the interaction between spike protein and the ACE-2 receptor [50]. An additional advantage of this new device is the development process and the optimization of conditions in BAU/mL, the international standard, in accordance with NIBSC (National Institute for Biological Standards and Control, UK) [51] or WHO-IS recommendations [52], which allows for further research to be conducted to compare the developed assay with other analytical methods. The LOD and assay time of the developed method are lower compared to commercial immunochromatographic tests that use colloidal gold. Additionally, the developed immunoassay enables a reduction in assay time when compared to an HRP-based VFIA (vertical flow immunoassay) because it eliminates the need for an incubation step with the substrate (Table 3).

A notable result of our study is the successful application of carbon black nanoparticles as labels in a VFIA. Previously, only one study reported the use of colloidal carbon in non-instrumental and semi-instrumental flow-through assays [19]. Despite the potential advantages, carbon black remains a less preferred label in lateral flow and flow-through tests, even though several studies have shown that it outperforms conventional gold nanoparticles [26,30,53]. We confirmed that commercial carbon black enables the preparation of antibody conjugates in a rapid, simple, and reproducible manner. Considering recent papers that demonstrate lower limits of detection (LODs) for other black-colored nanoparticles in LFIA [54], we suggest that researchers should pay more attention to CNPs as a commonly available and potentially more efficient alternative to conventional labels in point-of-care tests. At the same time, we identified several shortcomings associated with the use of CNPs. We observed a decrease in the functional activity of the conjugates during long-term storage, which may be due to the desorption of antibodies, both passively and as mediated by BSA present in the storage buffer. The non-uniform background is likely a result of hydrophobic interactions between carbon black nanoparticles and the nitrocellulose membrane. These interactions cannot be completely eliminated, even by coating the nanoparticles with an excess of proteins (IgG and BSA). One potential solution to overcome these disadvantages is the covalent attachment of antibodies [53] and the hydrophilization of CNP surfaces through polymer coating or chemical treatment.

**Table 3:** Comparison of the presented immunoassay with known methods of IgG vs. spike protein detection.

Assay	Label	LOD	Assay Time	Reference
Two-step CLIA *	Magnetic nanoparticles	12.16 BAU/mL	30–50 min	[55]
Rapid serological magnetic immunodetection	Magnetic nanoparticles	—	21 min	[56]
Immunochromatography (LFIA **)	Colloidal gold	30 BAU/ml	15 min	[57]
Immunochromatography (LFIA)	Colloidal gold	1:640	10 min	[58]
Immunochromatography (LFIA)	Colloidal gold	14.2 BAU/mL	10 min	[59]
Vertical flow immunoassay	Horseradish peroxidase	5 nM	15 min	[13]
Vertical flow immunoassay	Carbon nanoparticles	7.81 BAU/mL	5–7 min	This work

* CLIA—chemiluminescence immunoassay. ** LFIA—lateral flow immunoassay.

## Figures and Tables

**Table 1:** Assay reproducibility.

Serum Sample Number	ELISA, BAU/mL	Color Intensity, a.u. n = 6, Mean ± Standard Deviation	CV, %
3	5473.09	49 ± 3	6.1
63	2321.8	56.6 ± 3.7	6.5
36	1465.6	31.9 ± 2.6	8.1
83	564.9	21.6 ± 4.9	22.7
61	359.3	10.3 ± 1.6	15.1
58	108.0	11.7 ± 2.4	20.2
33	54.4	1.6 ± 2.3	145.8
48	20.4	0.4 ± 0.9	223.6
71	0	0.9 ± 1.9	223.6
75	0	2 ± 1.7	79.2

**Table 2:** Assay sensitivity and specificity rate.

Serum Sample Number	Detection Status for Visually Assessment	Sensitivity Rate, %	Specificity Rate, %
3	111111	100	0
63	111111	100	0
36	111111	100	0
83	111111	100	0
61	111111	100	0
58	011111	83	0
33	000000	0	100
48	000000	0	100
71	000000	0	100
75	000000	0	100

## Data Availability

The data presented in this study are available on request from the corresponding author.

## Supplementary Materials

The following supporting information can be downloaded at: https://www.mdpi.com/article/10.3390/bios13090857/s1, Figure S1: Fabrication of the vertical flow device.; Figure S2: SEM images of CNP@MAb; Figure S3: Scheme for the interpretation of assay results; Figure S4: The principle of obtaining an analytical signal using ImageJ software; Figure S5: Determination of IgG vs Spike-protein by the VFIA in optimal conditions; Table S1: Reproducibility of the CNP functionalization method; Table S2: Inter-operator precision.
