# Supplementary material for: Vertical Flow Immunoassay Based on Carbon Black Nanoparticles for the Detection of IgG against SARS-CoV-2 Spike Protein in Human Serum: Proof-of-Concept
Source: Biosensors (Basel). 2023 Aug 29;13(9):857. doi: 10.3390/bios13090857 (PMC10526127; doi:10.3390/bios13090857)
Supplement: Supplementary file 1 [file biosensors-13-00857-s001.zip › biosensors-2573195-supplementary.pdf]

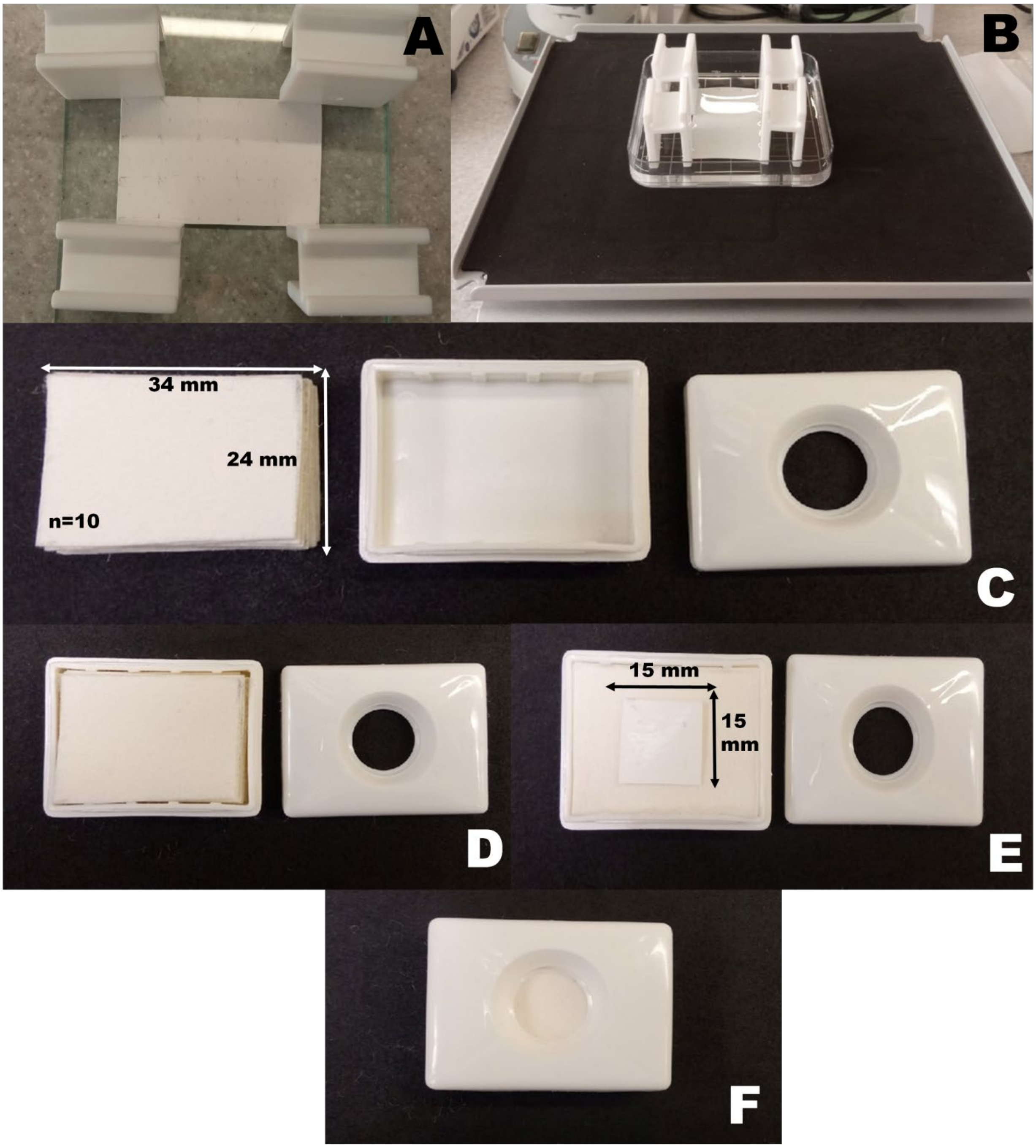

Figure S1. Fabrication of vertical flow device. A) Drying process of nitrocellulose strip where specific reagents are immobilized ; B) Washing /blocking process of nitrocellulose strip where specific reagents are immobilized; C, D) Disassembled plastic case with an absorbent pad; E) Disassembled vertical flow device with a nitrocellulose strip; F) Vertical flow device ready for use in immunoassay.

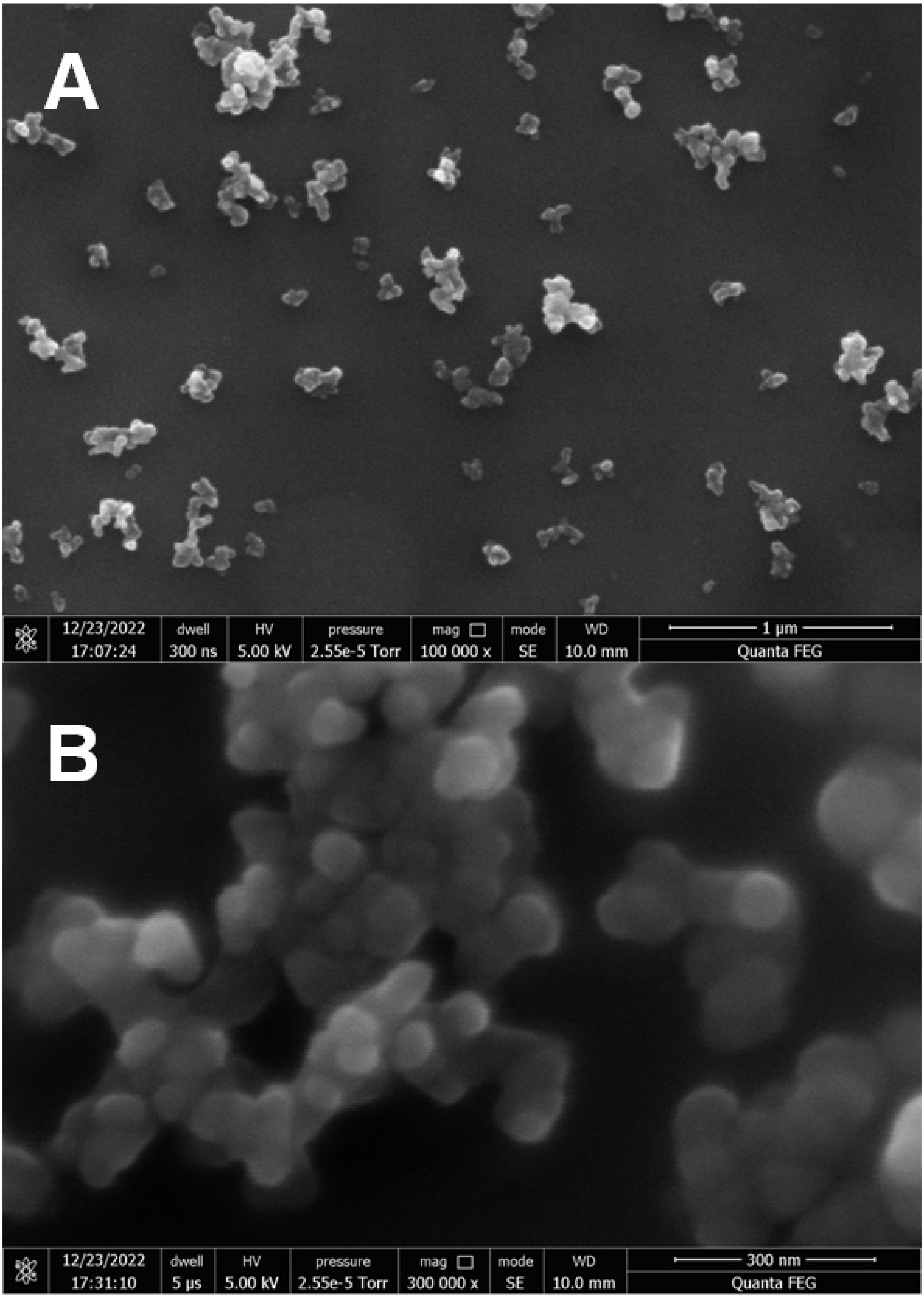

Figure S2. SEM images of CNP@MAb. Scale bar: A-1 µm; B-300 nm.

Table S1. Reproducibility of CNP functionalization method.

Conjugate number	Mean ± standard deviation	PDI	Concentration, mg/mL
№1	183<br>27.7	0.17<br>0.07	3.3
№2	182<br>15.8	0.19<br>0.07	3.5
№3	195<br>39.5	0.17<br>0.05	4.2

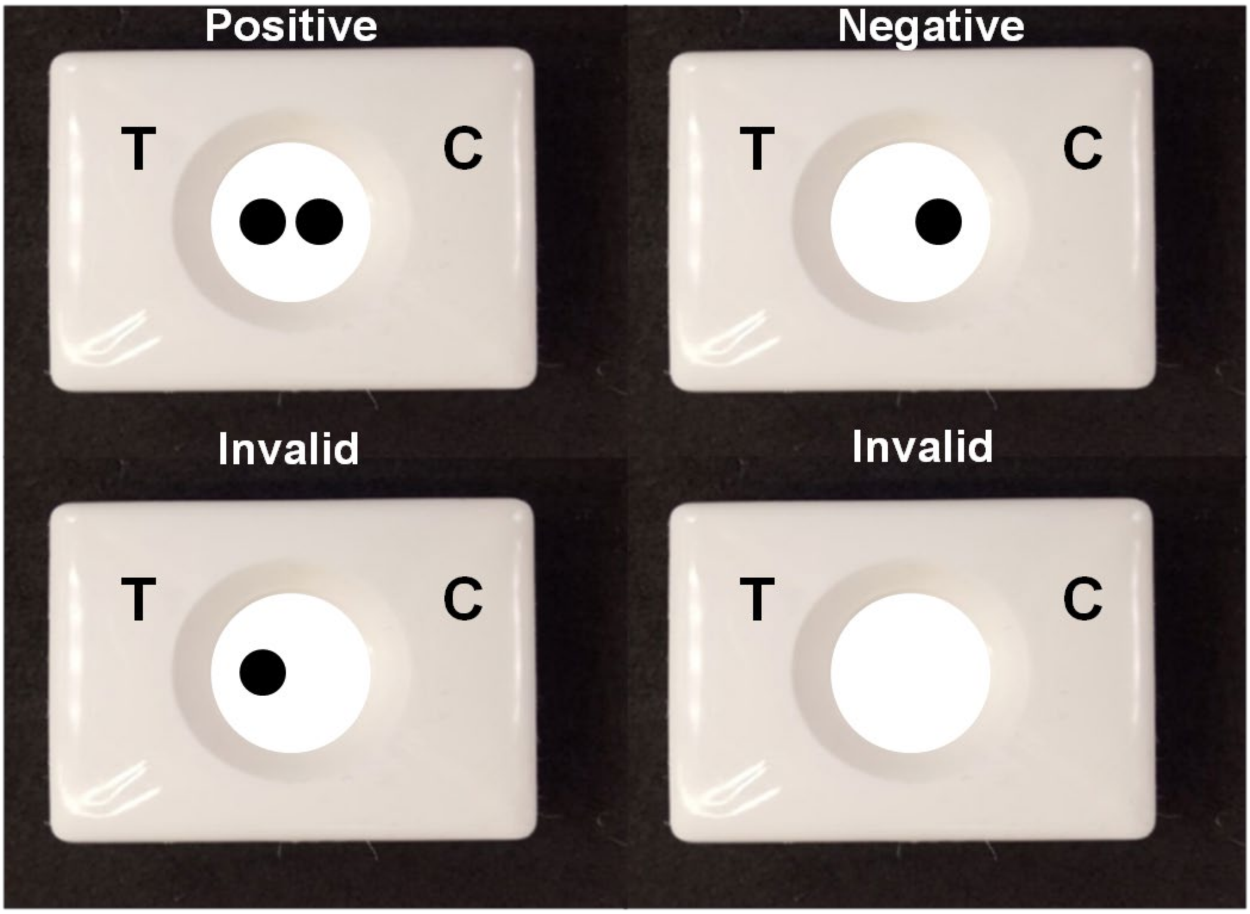

Figure S3. Scheme of assay results interpretation. C-control; T-test.

Table S2. Inter-operator precision.

Operator number	Visual assessment	Color intensity, a.u.	CV, %
Positive sample			
1	1	84.03	
2	1	89.9	
3	1	62,7	
4	1	85.2	
5	1	84.3	
6	1	74.9	
7	1	72.5	
8	1	83.65	
Mean± SD		79.54±8.32	10.44
Negative sample			
9	1	7.3	
10	0	-5.8	
11	0	-2.9	
12	0	-4.9	
13	0	-2.2	
14	?	-3.8	
15	0	-1.46	
16	0	0.917	

Quantitative reading of assay results with a flatbed scanner

We used a CanoScan Lide 600f flatbed scanner and CanoScan Toolbox 5.0 (Canon, Japan) to obtain digital images of the vertical flow assay results. To quantify the color of the spots, we have used the software ImageJ (National Institutes of Health, USA). The following scanning parameters were used: mode, grayscale image; resolution, 600 dpi, file format, JPEG. Then the gamma factor of the digital image was corrected in ImageJ to a value of 2 (ImageJ > Process > Math > Gamma). Using "Measure," we measured the color intensity of the background and spots in arbitrary units (AU) in the range from 255 (black) to 0 (white). Color intensity of the background was measured in 6 replicates and mean value was used. Then we have subtracted the spot intensity from mean background intensity. An example of analytical signal obtaining is shown in Fig S4.

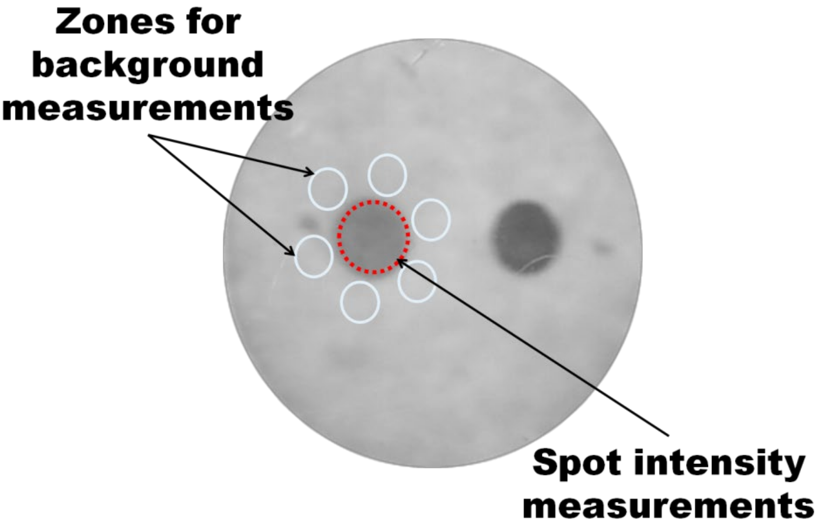

Figure S4. The principle of the obtaining an analytical signal using the ImageJ software.

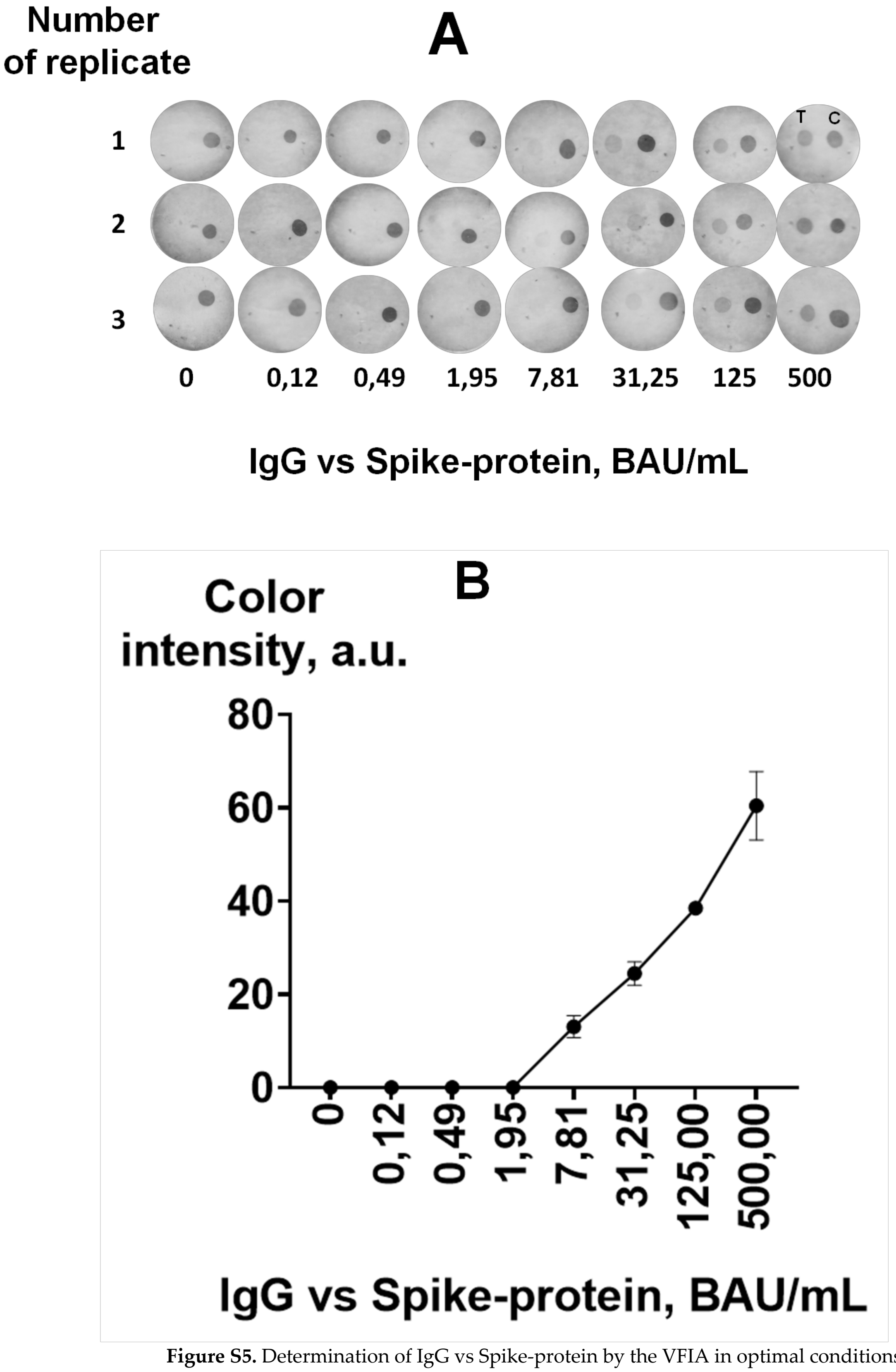

Figure S5. Determination of IgG vs Spike-protein by the VFIA in optimal conditions, n=3. A) Visual assessment of VFIA (T-test; C-control); B) Results obtained by scanner and ImageJ processing (the vertical bars indicate the standard deviation, n = 3).
